# Tailoring *Corynebacterium glutamicum* towards increased malonyl-CoA availability for efficient synthesis of the plant pentaketide noreugenin

**DOI:** 10.1186/s12934-019-1117-x

**Published:** 2019-04-11

**Authors:** Lars Milke, Nicolai Kallscheuer, Jannick Kappelmann, Jan Marienhagen

**Affiliations:** 1 Institute of Bio- and Geosciences, IBG-1: Biotechnology, Forschungszentrum Jülich, 52425 Jülich, Germany; 2 Bioeconomy Science Center (BioSC), Forschungszentrum Jülich GmbH, 52425 Jülich, Germany; 3 Institute of Biotechnology, RWTH Aachen University, Worringer Weg 3, 52074 Aachen, Germany

**Keywords:** Malonyl-CoA, *Corynebacterium glutamicum*, Noreugenin, Metabolic engineering, Acetyl-CoA carboxylase

## Abstract

**Background:**

In the last years, different biotechnologically relevant microorganisms have been engineered for the synthesis of plant polyphenols such as flavonoids and stilbenes. However, low intracellular availability of malonyl-CoA as essential precursor for most plant polyphenols of interest is regarded as the decisive bottleneck preventing high product titers.

**Results:**

In this study, *Corynebacterium glutamicum*, which emerged as promising cell factory for plant polyphenol production, was tailored by rational metabolic engineering towards providing significantly more malonyl-CoA for product synthesis. This was achieved by improving carbon source uptake, transcriptional deregulation of *accBC and accD1* encoding the two subunits of the acetyl-CoA carboxylase (ACC), reduced flux into the tricarboxylic acid (TCA) cycle, and elimination of anaplerotic carboxylation of pyruvate. The constructed strains were used for the synthesis of the pharmacologically interesting plant pentaketide noreugenin, which is produced by plants such as *Aloe arborescens* from five molecules of malonyl-CoA. In this context, accumulation of the C_1_/C_6_ cyclized intermediate 1-(2,4,6-trihydroxyphenyl)butane-1,3-dione (TPBD) was observed, which could be fully cyclized to the bicyclic product noreugenin by acidification.

**Conclusion:**

The best strain *C. glutamicum* Nor2 C5 mu*fasO*_*BCD1*_ P_O6_-*iolT1* ∆*pyc* allowed for synthesis of 53.32 mg/L (0.278 mM) noreugenin in CGXII medium supplemented with casamino acids within 24 h.

**Electronic supplementary material:**

The online version of this article (10.1186/s12934-019-1117-x) contains supplementary material, which is available to authorized users.

## Background

Besides alkaloids and isoprenoids, polyphenols constitute the third large group of plant secondary metabolites [1]. In plants, polyphenols such as flavonoids, stilbenes or lignans do not contribute directly to growth or propagation, they rather protect the plant from UV radiation, aid in the defense against pathogens or herbivores, and color petals and fruits to attract animals [2, 3]. Due to anti-oxidative, anti-depressive, anti-hepatotoxic, anti-cancerous as well anti-inflammatory effects described for many polyphenols, these compounds received a lot of attention over the last years [4–8].

Recently, the Gram-positive soil bacterium *Corynebacterium glutamicum* was engineered towards plant polyphenols synthesis [9, 10]. Important prerequisite for establishing *C. glutamicum* as polyphenol producing cell factory was the identification and abolishment of a catabolic phenylpropanoid pathway. To date, several stilbenes (e.g. resveratrol and its *O*-methylated derivatives) as well as flavonoids (e.g. naringenin, kaempferol and quercetin) could be produced with *C. glutamicum* from supplemented phenylpropanoid precursors or directly from glucose [10, 11]. In the context of these studies, low intracellular availability of malonyl-CoA provided by the central metabolism could be identified as major bottleneck impeding higher product titers [2]. When considering the stoichiometry of flavonoid and stilbene synthesis, however, the importance of larger amounts of malonyl-CoA for plant polyphenol synthesis is not surprising as three moles of malonyl-CoA are consumed by chalcone synthases (CHS) and stilbene synthases (STS) during condensation with a CoA-activated phenylpropanoid thioester yielding chalcones (as flavonoid precursors) and stilbenes, respectively [12]. Similar to engineered polyphenol production in other microorganisms, the observed limitation at the stage of malonyl-CoA could be overcome in *C. glutamicum* at lab-scale by supplementing the antibiotic cerulenin, which selectively inhibits fatty acid synthesis as main malonyl-CoA sink in the microbial carbon metabolism [10, 13, 14]. Very recently, dependency on cerulenin for plant polyphenol synthesis could be repealed by rationally engineering the central metabolism yielding the *C. glutamicum* C7 strain, which enabled the accumulation of 24 mg/L (0.088 mM) naringenin or 112 mg/L (0.49 mM) resveratrol from glucose [15]. Key to success was a reduction of the flux into the tricarboxylic acid (TCA) cycle, whereas episomal overexpression of *accBC* (cg0802) and *accD1* (cg0812) encoding the two subunits of the acetyl-CoA carboxylase complex (ACC) hardly increased product titers. In *C. glutamicum*, transcription of *accBC* and *accD1* is controlled by the transcriptional repressor FasR [16]. Hence, *fasR* (cg2737) was also deleted to deregulate expression of *accBC* and *accD1*, but the observed positive effect on malonyl-CoA availability and accumulation of the flavonoid naringenin was very limited [15]. This was explained by the previous finding that FasR also represses expression of *fas*-IA (cg2743) and *fas*-IB (cg0957) coding for the two fatty acid synthases of *C. glutamicum*, which consume malonyl-CoA provided by the ACC for fatty acid biosynthesis [16].

**Fig. 1:**
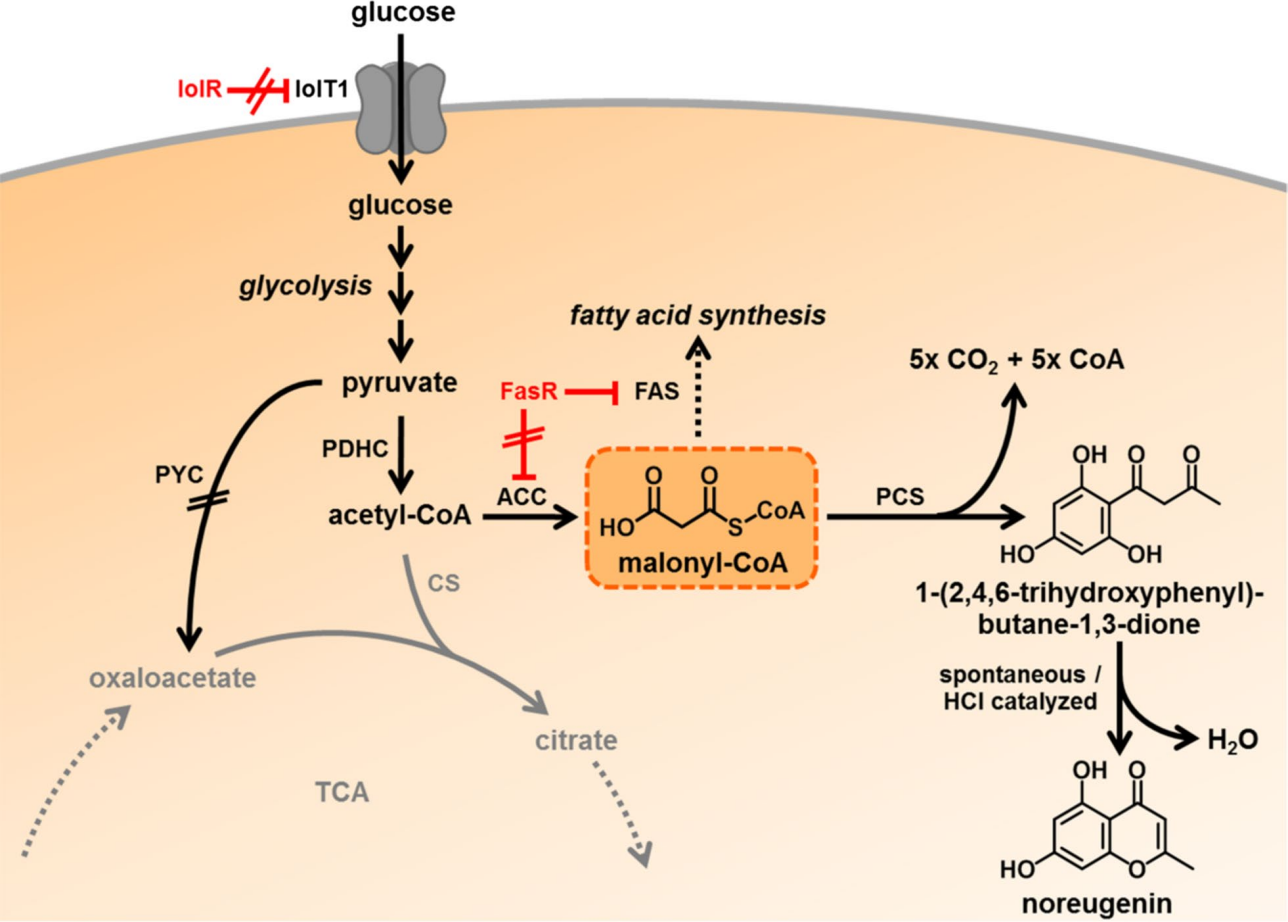
Metabolic engineering of the central carbon metabolism of *C. glutamicum* for increased malonyl-CoA availability and noreugenin synthesis. ACC: acetyl-CoA carboxylase; CS: citrate synthase; FAS: fatty acid synthase; FasR: transcriptional repressor of *accBC*, *accD1*, *fasIA* and *fasIB*, all involved in fatty acid synthesis; IolR: transcriptional repressor of genes involved in *myo*-inositol catabolism; IolT1: glucose/*myo*-inositol permease; PCS: pentaketide chromone synthase; PDHC: pyruvate dehydrogenase complex; PYC: pyruvate carboxylase; TCA: tricarboxylic acid cycle, Eliminated enzymatic reactions (black) or regulatory circuits (red): crossed out; reduced enzyme/pathway activity: grey

About a decade ago, the pentaketide chromone synthase (PCS_*Aa*_, EC 2.3.1.216, UniProt ID: Q58VP7) was identified in the medicinal plant *Aloe arborescens* [17]. Like CHS- and STS-enzymes, PCS_*Aa*_ is a type III polyketide synthase (PKS), but instead of catalyzing the condensation of three malonyl-CoA extender units with a CoA-activated phenylpropanoid thioester, this enzyme catalyzes the iterative decarboxylation and condensation of five malonyl-CoA molecules yielding the chromone noreugenin (Fig. 1). Plant-derived chromones such as noreugenin are gaining attention drawn by their beneficial impacts on human health including anti-inflammatory, anti-cancerous, anti-diabetic but also antimicrobial traits [18]. Thus, the chromone scaffold is considered to be a promising lead structure for medicinal chemistry. Noteworthy, the furochromones khellin and visnagin, both regarded to have anti-asthmatic effects, are derived from the pentaketide noreugenin [19]. Chemical routes for the synthesis of noreugenin include acid- or base-catalyzed reactions, microwave irradiation and solid-support catalysts [20, 21]. In addition to chemical synthesis, noreugenin could also be obtained by extraction from the producing plant material. However, typically, chemical synthesis of more complex secondary plant metabolites is economically not feasible and product concentrations in the naturally producing plants are usually quite low. In this context, microbial production of secondary plant metabolites has emerged as true alternative [22]. In case of noreugenin, synthesis of this compound has been first described in in vitro enzyme assays with purified PCS_*Aa*_ [17]. Recently, microbial noreugenin synthesis in *Escherichia coli* using PCS_*Aa*_ has been reported for the first time as proof of principle, but no product titer was determined [23].

Here, we present rational engineering of the central carbon metabolism and of fatty acid synthesis in *C. glutamicum* towards further increasing malonyl-CoA availability. Furthermore, we demonstrate the functional integration of PCS_*Aa*_ into these engineered *C. glutamicum* strains and the microbial synthesis of noreugenin.

## Results

### Establishing a heterologous pathway for the synthesis of noreugenin

**Fig. 2:**
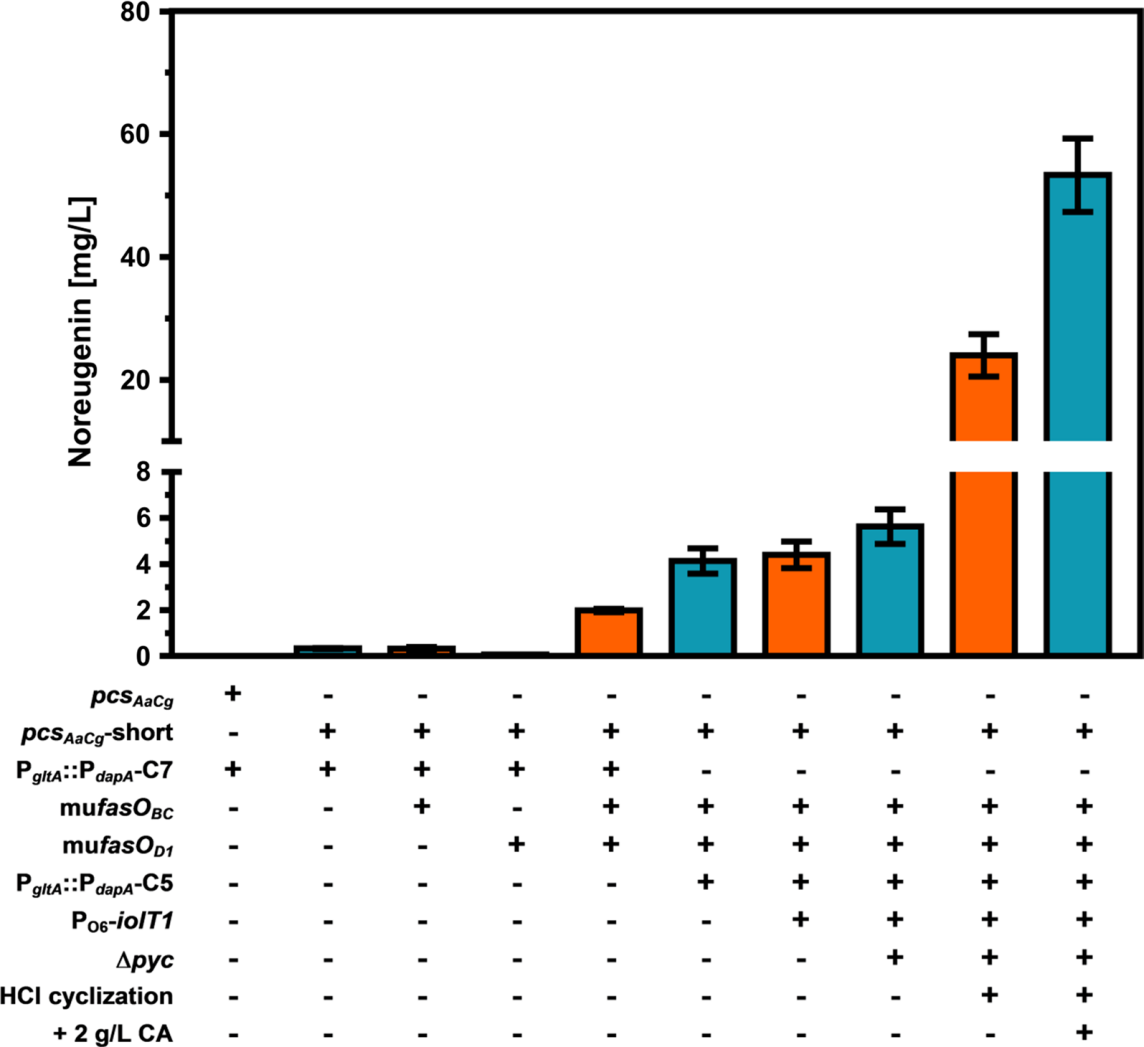
Overview of the constructed *C. glutamicum* strains for improved intracellular malonyl-CoA availability and noreugenin synthesis. Given noreugenin concentrations were determined after 72 h of cultivation and represent mean values with standard deviations from biological triplicates. For more clarity, + and - represent presence or absence of the depicted genetic modifications or plasmids. For details the reader is referred to the main text

For establishing noreugenin synthesis in *C. glutamicum*, a codon-optimized, synthetic variant of the *pcs* gene (*pcs*_*AaCg*_) originating from *A. arborescens* was cloned into the vector pMKEx2 yielding pMKEx2-*pcs*_*AaCg*_, which allows for isopropyl β-d-thiogalactopyranoside (IPTG) inducible gene expression of *pcs*_*AaCg*_ from the strong T7 promoter [24]. After transformation of *C. glutamicum* C7 selected as production strain due to its improved capability to supply malonyl-CoA, the resulting strain *C. glutamicum* C7 pMKEx2-*pcs*_*AaCg*_ (designated *C. glutamicum* Nor1 C7) was cultivated in defined CGXII minimal medium with 4% glucose and 1 mM IPTG. However, these initial experiments yielded only traces of noreugenin close to the detection limit of our LC–MS system (Fig. 2).

Detailed comparison of the amino acid sequence of PCS_*Aa*_ to similar type III PKS such as the STS from *Arachis hypogea* (STS_*Ah*_) and CHS from *Petunia x hybrida* (CHS_*Ph*_) revealed that the first ten amino acids of PCS_*Aa*_ have no equivalent sequence in any of the two other enzymes, despite their otherwise high sequence identity of 70–80% over the whole amino acid sequence (Additional file 1: Figure S1). Surprisingly, residues M11 and V14 of PCS_*Aa*_ align with the starting methionine M1 and V4 of both enzymes, CHS_*Ph*_ and STS_*Ah*_. This pointed either to a simple misannotation of the translational start or to the ten N-terminal amino acids of PCS_*Aa*_ functioning as signal sequence for protein localization in the plant cell. Three algorithms (SignalP [25], TargetP [26] and WoLF PSORT [27]) developed to detect signal- and targeting peptide sequences were used to interpret this sequence, but none proposed a possible function for this short amino acid stretch. However, a pMKEx2 plasmid harboring a truncated *pcs*_*AaCg*_ variant (*pcs*_*AaCg*_-short, pMKEx2-*pcs*_*AaCg*_-short) without the first 30 nucleotides encoding the ten N-terminal amino acids in question was constructed. Expression of the truncated *pcs*_*AaCg*_ gene in the strain *C. glutamicum* C7 (designated *C. glutamicum* Nor2 C7) under aforementioned conditions, significantly improved noreugenin synthesis leading to a product titer of 0.8 mg/L (0.004 mM) after 72 h (Fig. 2). Hence, most likely the *pcs*_*Aa*_ was simply not annotated correctly, likely reducing the activity of the enzyme or preventing the formation of the active PCS_*Aa*_ dimer under the described conditions.

### Deregulation of genes involved in fatty acid synthesis increases malonyl-CoA availability

**Fig. 3:**
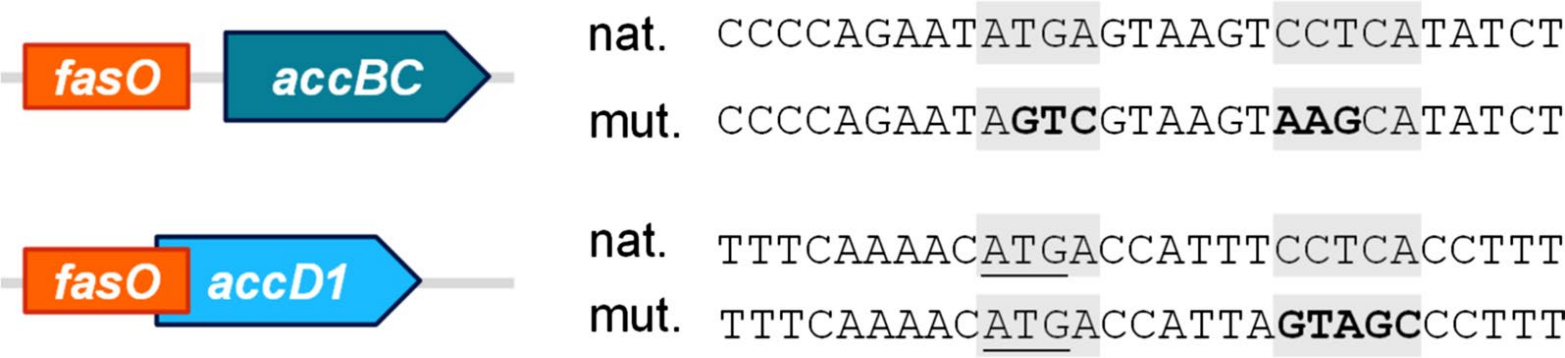
Schematic representation and relative postion of the *fasO* sites of *accBC* and *accD1*. In both cases, the mutated (mut.) *fasO* sites are aligned with the respective native (nat.) *fasO* sites. Grey-backed nucleotides are conserved in all known *C. glutamicum fasO*-sites essential for FasR binding. Mutated nucleotides are highlighted by bold letters. The start codon of *accD1* is underlined

In order to further improve noreugenin synthesis, fatty acid metabolism of *C. glutamicum* Nor2 C7 was engineered aiming for increased malonyl-CoA supply. The fatty acid regulator protein FasR acts as transcriptional repressor of the genes *accBC*, *accD1*, *fasIA* and *fasIB,* all involved in fatty acid biosynthesis [16]. Deletion of *fasR* in *C. glutamicum* C7 was shown to negatively affect flavonoid synthesis, presumably due to an also increased fatty acid synthase (FAS) activity consuming malonyl-CoA again [15]. In contrast, episomal overexpression of ACC subunit genes *accBC* and *accD1* increased the polyphenol production by 40%, which shows that acetyl-CoA carboxylation is indeed a promising target for further metabolic engineering. Therefore, fatty acid metabolism of *C. glutamicum* should be tailored towards increased endogenous ACC activity yielding more malonyl-CoA without simultaneously increasing FAS activity consuming this noreugenin precursor. Mutational analysis of the operator regions of both *acc* genes identified the *fasO* motifs essential for FasR binding [16]. Based on these findings, the *fasO* motifs upstream of the *accBC*- and *accD1*- open reading frames were mutated in the *C. glutamicum* C7 strain background, both individually (mu*fasO*_*BC*_, mu*fasO*_*D1*_) and in combination (mu*fasO*_*BCD1*_). Important to note in this context, since the FasR binding site of *accD1* overlaps with the open reading frame of this gene, only nucleotide substitutions were introduced that do not alter the amino acid sequence of AccD1 (Fig. 3). Subsequently, the resulting strains *C. glutamicum* Nor2 C7 mu*fasO*_*BC*_, *C. glutamicum* Nor2 C7 mu*fasO*_*D1*_ and *C. glutamicum* Nor2 C7 mu*fasO*_*BCD1*_ were cultivated to compare their ability to produce noreugenin (Fig. 2). Whereas the strains with individually mutated *fasO* sites accumulated 0.31 mg/L (0.002 mM) and 0.06 mg/L (0.0003 mM), respectively, did *C. glutamicum* Nor2 C7 mu*fasO*_*BCD1*_ generate 1.98 mg/L (0.010 mM) noreugenin after 72 h of cultivation in CGXII medium with 4% glucose.

**Fig. 4:**
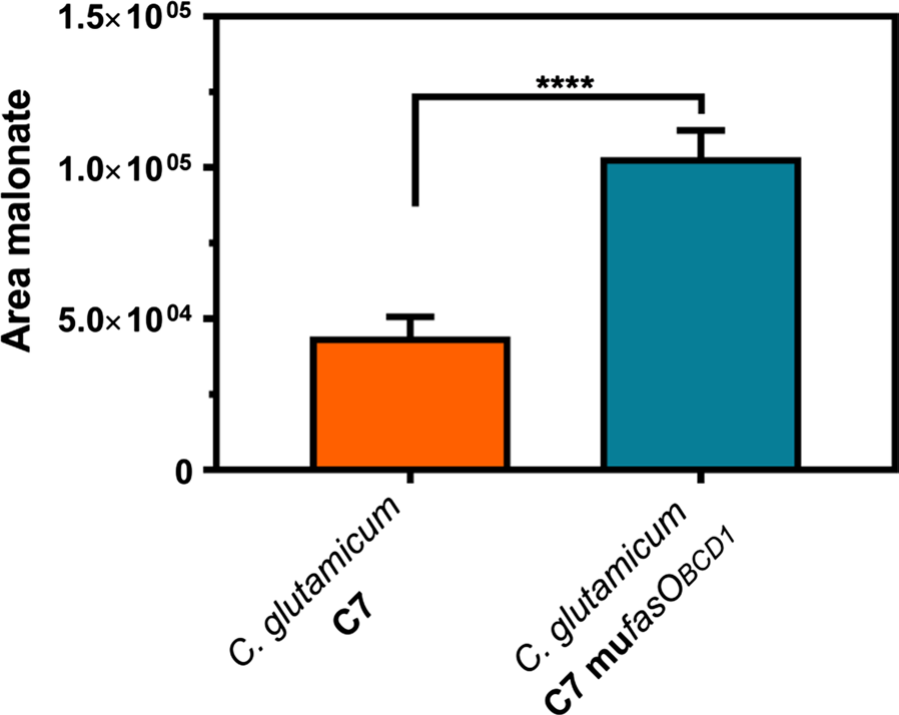
LC–MS/MS analysis of intracellular malonate concentrations representing intracellular malonyl-CoA availability. Areas for malonate acquired by LC-MS/MS analysis of cytoplasmatic extracts are plotted for the indicated strains. The obtained malonate areas represent mean values with standard deviations from biological duplicates with three technical replicates each (six samples in total). The four asterisks represent the level of significance of an unpaired two-tailed *t* test (p < 0.0001)

With the aim of verifying that *C. glutamicum* C7 mu*fasO*_*BCD1*_ indeed provides more malonyl-CoA compared to *C. glutamicum* C7, intracellular malonyl-CoA was quantified in form of its free acid malonate by LC–MS/MS as previously described [15]. As the expression of *pcs*_*AaCg*_-short would lead to malonyl-CoA consumption and thus distort intracellular metabolite concentrations, the strains without the pMKEx2-*pcs*_*AaCg*_-short plasmid were examined. Since the obtained isotope ratio for the parental *C. glutamicum* C7 strain was below the limit of quantification, obtained signal areas themselves were used for comparison (Fig. 4). The malonate area for the parental strain *C. glutamicum* C7 was determined to be 43,065 ± 3090, whereas the malonate signal for *C. glutamicum* C7 mu*fasO*_*BCD1*_ was increased 2.8-fold (102,304 ± 4495). As the isotope ratio corresponding to the latter area was within the linear range of the calibration curve, an intracellular malonate concentration of 1.8 mM for *C. glutamicum* C7 mu*fasO*_*BCD1*_ could be calculated. These findings show that noreugenin synthesis can be used to quickly report differences in intracellular malonyl-CoA pools of constructed *C. glutamicum* strains, instead of always using the laborious LC–MS/MS-based method. This allows considering noreugenin as a reliable reporter molecule to evaluate the impact of metabolic engineering on intracellular malonyl-CoA availability in addition to the relevance of the product itself.

### Increased glucose uptake and reduced flux into the tricarboxylic acid cycle increase the malonyl-CoA-pool

Previously, reduction of the activity of citrate synthase (CS, encoded by the gene *gltA*, cg0949) to 10% compared to wild-type CS activity by promoter replacement enabled increased plant polyphenol production with *C. glutamicum* due to a reduced flux of acetyl-CoA in the TCA cycle and thus increased malonyl-CoA availability [15]. Further reduction of CS activity to 5.5% was achieved by exchanging the P_*dapA*_-C7 promoter upstream of *gltA* by the even weaker P_*dapA*_-C5 variant [15, 28]. The constructed strain *C. glutamicum* Nor2 C5 mu*fasO*_*BCD1*_ showed reduced growth, but accumulated two times more noreugenin after 72 h of cultivation (4.13 mg/L, 0.022 mM) compared to *C. glutamicum* Nor2 C7 mu*fasO*_*BCD1*_ (1.98 mg/L, 0.010 mM, Fig. 2).

The dependency of noreugenin synthesis on the availability of the glucose-derived metabolite malonyl-CoA suggests that an improved glucose uptake of *C. glutamicum* might further increase the intracellular malonyl-CoA pool. Recently, we reported that relieve of IolR-mediated repression of the *iolT1* gene encoding the glucose/*myo*-inositol permease IolT1 by promoter engineering increases d-xylose and d-glucose uptake in *C. glutamicum* [29, 30]. Hence, two point mutations in the *iolT1* promoter (P_O6_-*iolT1*) were also introduced into *C. glutamicum* Nor2 C5 mu*fasO*_*BCD1*_ yielding *C. glutamicum* Nor2 C5 mu*fasO*_*BCD1*_ P_O6_-*iolT1*. Interestingly, this modification appears to have only a minor positive effect on noreugenin synthesis (4.40 mg/L, 0023 mM, Fig. 2), but growth, negatively influenced by downregulation of *gltA* expression, was partly improved.

To further increase acetyl-CoA availability for malonyl-CoA synthesis, anaplerotic reactions withdrawing phosphoenolpyruvate (PEP) and pyruvate as glycolytic acetyl-CoA precursors were targeted. Interestingly, *C. glutamicum* is capable of catalyzing the carboxylation of both PEP and pyruvate during growth on glucose, which is rather uncommon for most microorganisms [31]. It was demonstrated, that PEP carboxylase (PEPC) and pyruvate carboxylase (PYC) could replace each other as anaplerotic enzymes when glucose is used as sole carbon and energy source [32]. This means that at least one of the two enzymes needs to be present for enabling growth on glucose. In fact, 90% of total oxaloacetate synthesis is ascribed to the activity of PYC encoded by *pyc* (cg0791) in *C. glutamicum* [33]. Therefore, we decided to delete *pyc.* The strain *C. glutamicum* Nor2 C5 mu*fasO*_*BCD1*_ P_O6_-*iolT1* ∆*pyc* was constructed, cultivated and characterized with regard to noreugenin synthesis. Deletion of the *pyc* gene in this strain increased noreugenin synthesis and enabled accumulation of 5.63 mg/L (0.029 mM) noreugenin within 72 h (Fig. 2).

### Identification and acid-catalyzed cyclization of the intermediate 1-(2,4,6-trihydroxyphenyl)butane-1,3-dione

**Fig. 5:**
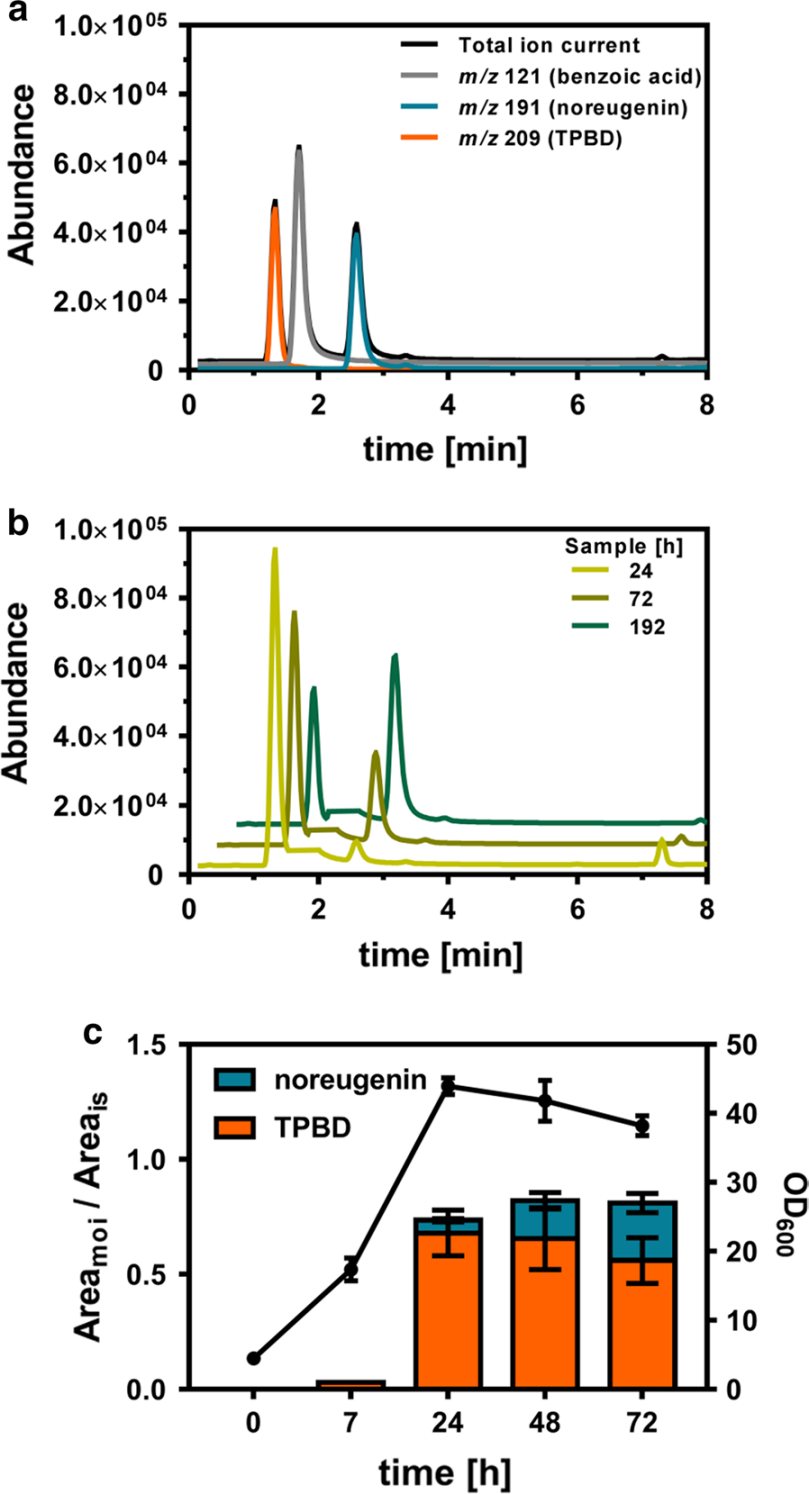
Detection of the noreugenin synthesis intermediate 1-(2,4,6-trihydroxyphenyl)butane-1,3-dione (TPBD) in extracted cultivation samples. **a** Exemplary chromatogramm of an extracted cultivation sample both as total ion current and the individual *m/z* ratios. **b** Total ion current chromatogramms of extracted samples resembling the spontaneous cyclization of TPBD towards noreugenin over the cultivation time. For better visualization, the benzoic acid signal was removed from the chromatogramm. **c** Growth curve and product abundance for the strain *C. glutamicum* C5 *fasO*_*BCD1*_ P_O6_-*iolT1* ∆*pyc* pMKEx2-*pcs*_*AaCg*_-short. Unavailability of an authentic TPBD standard prevented quantification of this particular molecule. To evaluate product formation over time, the normalized signal areas of the molecules of interest (Area_moi_) TPBD and noreugenin were added up. Signal area of the internal standard (Area_is_) benzoic acid was used for normalization. The calculated ratios are depicted on the primary Y-axis. At the given sampling time points, OD_600_ of the cultures was also determined (filled circles, shown on secondary Y-axis). The obtained data represent mean values with standard deviations from biological triplicates

To our surprise, synthesis of noreugenin continued over the whole process time in all *C. glutamicum* strains constructed in this study, although the cells reached the stationary growth phase already after 24 h. To the best of our knowledge, malonyl-CoA supply in *C. glutamicum* is strictly coupled to the exponential growth phase, implying that noreugenin synthesis should cease when the cultures reach the stationary growth phase. In preceding studies focusing on the synthesis of malonyl-CoA-dependent polyphenols naringenin and resveratrol with *C. glutamicum* C7-based strains, continuous production of the respective polyphenols was never observed [15]. Even when prolonging the cultivation times from 72 to 192 h, noreugenin formation continued and did not reach a plateau (Additional file 1: Figure S2). We assumed that this phenomenon was unlikely to be due to the engineered *C. glutamicum* strain background, instead we concluded that the reason for this finding must be connected to the product noreugenin itself. As noreugenin synthesis from malonyl-CoA is catalyzed by a single enzyme, further investigations focused on PCS_*Aa*_ and its reaction mechanism. PCS_*Aa*_ is described to catalyze a C_1_/C_6_ Claisen-type cyclization of the enzyme-bound pentaketide followed by spontaneous pyrone ring formation yielding noreugenin [34]. In silico reconstruction of the PCS_*Aa*_ reaction mechanism suggested 1-(2,4,6-trihydroxyphenyl)butane-1,3-dione (TPBD, M = 210,05 g/mol) to be the C_1_/C_6_ cyclized pentaketide intermediate that subsequently undergoes spontaneous isomerization and dehydration (Fig. 1). When the mass spectrometer was operated in negative electrospray ionization (ESI) mode and data acquisition was performed in selected ion monitoring (SIM) mode, an additional mass signal at *m/z* 209 (retention time = 1.33 min) was detected in all prepared samples representing the [M−H]^−^ mass signal for the presumed intermediate TPBD (Fig. 5a). Over the cultivation time, a decreasing TPBD signal could be observed whereas the noreugenin signal increased (Fig. 5b). Unfortunately, unavailability of an authentic TPBD standard rendered quantification of this intermediate impossible. To evaluate product formation over time, we added up the signal areas of TPBD and noreugenin, both normalized to the area of the internal standard benzoic acid ((Area_Noreugenin_/Area_Benzoic acid_) + (Area_TPBD_/Area_Benzoic acid_)) (Fig. 5c). Here, no net change of summed ratios beyond 24 h of cultivation could be observed. We concluded, that the synthesized TPBD circularizes spontaneously after this time point, forming the pyrone moiety yielding noreugenin. This would explain the observed decreasing TPBD concentration and an increasing noreugenin concentration over time. However, since the summed ratio of TPBD and noreugenin does not change after 24 h, no more TPBD is synthesized when the cells have reached the stationary growth phase and malonyl-CoA supply stopped after 24 h when glucose is depleted. Therefore, the observed continuous noreugenin synthesis can solely be ascribed to slow, spontaneous TPBD conversion and is not due to any unlikely growth-decoupled supply of malonyl-CoA.

As the slow, spontaneous cyclization of the pyrone moiety limits the absolute noreugenin titer and needlessly prolongs the overall process time, speeding up TPBD conversion was targeted to fully convert TPBD to noreugenin. For this purpose, acetonitrile extracts prepared for LC–MS analysis were acidified with methanolic HCl at various concentrations. After evaporation to dryness and subsequent resuspension in acetonitrile, conversion of TPBD to noreugenin was quantified using LC–MS analysis. These experiments showed, that a concentration of at least 142 mM HCl was required to achieve full conversion of TPBD to noreugenin (data not shown). Here, we proposed an acid-catalyzed mechanism for the cyclization of TPBD (Additional file 1: Figure S3). Application of this method for samples obtained from standard cultivation of the best performing strain *C. glutamicum* Nor2 C5 mu*fasO*_*BCD1*_ P_O6_-*iolT1* ∆*pyc* increased the total noreugenin titer to 23.99 mg/L (0.125 mM, Fig. 2).

### Supplementation of defined CGXII medium with casamino acids

Recently, standard CGXII medium was described to be insufficient to support heterologous expression of the plant-derived genes *ans* and *3gt* encoding an anthocyanidin synthase and an anthocyanidin 3-*O*-glucosyltransferase required for anthocyanin biosynthesis in *C. glutamicum* [35]. In this context, utilization of Andrew’s magic medium (AMM), described as a rich medium for *E. coli* already containing 2 g/L casamino acids, was tested [36]. Additional supplementation of 2 g/L casamino acids for improving the functional expression of these genes allowed for higher anthocyanin titers with *C. glutamicum* from supplemented catechin. With the aim to exclude that microbial noreugenin synthesis with *C. glutamicum* Nor2 C5 mu*fasO*_*BCD1*_ P_O6_-*iolT1* ∆*pyc* suffers from poor expression of *pcs*_*AaCg*_-short due to an unfavorable medium composition, standard CGXII medium with 4% glucose was additionally supplemented with 2 g/L casamino acids. By doing so, noreugenin synthesis could be doubled yielding 53.32 mg/L (0.278 mM) after 72 h of cultivation and subsequent HCl-based cyclization of the TPBD intermediate (Fig. 2).

## Discussion

In this study, we describe engineering of *C. glutamicum* for increased malonyl-CoA availability and microbial synthesis of the plant pentaketide noreugenin originally found in the medicinal plant *A. arborescens*. Key to success was the N-terminal truncation of the type III PKS enabling noreugenin synthesis. Originally, the ten N-terminal residues of PCS_*Aa*_ were believed to contribute to the formation of an expanded surface-exposed loop [34]. However, since this short amino acid stretch is not present in closely related CHS- and STS-enzymes, which could be already functionally implemented in *C. glutamicum*, we deleted the first 30 nucleotides of the *pcs*_*Aa*_ gene. Eventually, this modification drastically improved noreugenin synthesis.

A common approach in the context of improving malonyl-CoA availability for product synthesis aims at increasing ACC activity in the respective microbial workhorse [2]. Previously, by abolishing Snf1-dependent posttranslational regulation of Acc1, malonyl-CoA availability in *Saccharomyces cerevisiae* could be increased for the synthesis of fatty acid ethyl esters and 3-hydroxypropionic acid [37]. Another strategy for increasing ACC activity is overexpression of genes encoding for the respective subunits of this enzyme complex. For example, episomal overexpression of four genes coding for a heterotetrameric ACC from *Photorhabdus luminescens* in *E. coli* increased the titer of the malonyl-CoA-derived polyphenol pinocembrin sevenfold, yielding 196 mg/L of this product [38]. Furthermore, the active AccBC-AccD1 heterodimer of *C. glutamicum* could be functionally introduced into *E. coli* [39]. By following this strategy, the intracellular malonyl-CoA availability in *E. coli* was increased 15-fold allowing for the synthesis of 1.3 g/L phloroglucinol [40]. In this study, we developed a different strategy aiming for an increased expression of the genomically encoded ACC genes in *C. glutamicum*, instead of following the more traditional approach of episomal overexpression of (heterologous) genes. By mutation of the *fasO*-sites upstream of *accBC* and *accD1*, repression of gene expression mediated by the transcription regulator FasR could be repealed, which almost tripled intracellular malonyl-CoA availability and thus enabled increased product formation. Important to note, mutation of both FasR binding sites was required to increase ACC activity, probably because a functional ACC requires both subunits, AccBC and AccD1, in equimolar amounts [39, 41]. Furthermore, the intracellular malonyl-CoA availability was positively influenced by increasing glucose uptake through deregulation of the gene for the glucose/*myo*-inositol permease IolT1. However, this had only limited beneficial effects on noreugenin synthesis at shaking flask scale, but might be beneficial for large-scale applications when considering important process criteria such as space–time yield. Efficacy of this strain modification was already demonstrated for the de novo synthesis of hydroxybenzoic acids in *C. glutamicum* from glucose [30]. Potential other strategies for increasing the intracellular malonyl-CoA availability include establishing an ACC-independent pathway for malonyl-CoA synthesis from supplied malonate through heterologous expression of genes for malonate uptake and CoA-activation of malonate originating from the malonate assimilation pathway in *Rhizobium trifolii* [42]. This particular approach enabled a 15-fold increase of pinocembrin production using *E. coli* [13, 43]. Furthermore, state-of-the-art techniques for gene silencing or downregulation such as CRISPR interference (CRISPRi) or methods employing synthetic small regulatory RNA (sRNA) libraries or synthetic antisense RNA (asRNA) could be used to specifically knock down genes encoding for malonyl-CoA consuming enzymes [23, 44, 45].

Moreover, we predicted and detected the TPBD intermediate as the actual product of PCS_*Aa*_ and could show that formation of TPBD is strictly limited to the exponential growth phase in *C. glutamicum* in which malonyl-CoA is exclusively supplied. This is in line with our observations regarding flavonoid- and stilbene synthesis with *C. glutamicum* [10, 15]. Through HCl acidification of acetonitrile extracts we achieved full conversion of TPBD to noreugenin within 3 h allowing to significantly reduce the overall cultivation time.

Very recently, it could be demonstrated that composition of the defined CGXII medium might not be optimal for the expression of plant-derived genes involved in anthocyanin synthesis in *C. glutamicum* [35]. In this particular study, supplementation of the defined AMM medium with 2 g/L casamino acids significantly improved heterologous gene expression and thus anthocyanin synthesis. Casamino acids, obtained through acid hydrolysis of casein, represent a valuable source of all proteinogenic amino acids except tryptophan [46, 47]. Although it could be shown here that supplementation of casamino acids also promotes noreugenin synthesis, we prefer the simplicity of defined CGXII medium, especially as the composition of casamino acids varies from supplier to supplier. Nevertheless, this supplementation strategy is helpful to meet possible future challenges connected to heterologous gene expression. Alternatively, translational fusions of the target protein with the maltose-binding protein MalE from *E. coli* can be generated. This strategy already proved to be beneficial for the functional expression of a plant-derived *O*-methyltransferase gene from *Vitis vinifera* in *C. glutamicum* [11].

## Conclusion

In the present work, we applied the knowledge of the well-characterized central carbon metabolism to tailor *C. glutamicum* towards increased malonyl-CoA availability by rational metabolic engineering. Additionally, our work contributes to a better understanding of the PCS_*Aa*_ reaction mechanism as we could detect the intermediate TPBD, which spontaneously cyclizes to noreugenin. Acidification accelerated TPBD conversion to the product allowing for a titer of 53.32 mg/L (0.278 mM) noreugenin. Taken together, the constructed strain *C. glutamicum* C5 mu*fasO*_*BCD1*_ P_O6_-*iolT1* ∆*pyc* represents a promising strain for the microbial production of noreugenin and other malonyl-CoA derived products.

## Materials and methods

### Bacterial strains, plasmids, media and growth conditions

All bacterial strains and plasmids with their respective characteristics used in this study are listed in Table 1. *C. glutamicum* strains were routinely cultivated aerobically at 30 °C in brain heart infusion (BHI) medium (Difco Laboratories, Detroit, USA) or defined CGXII medium with glucose as sole carbon and energy source [48]. When indicated, 2 g/L casamino acids were supplemented (Becton–Dickinson, Franklin Lakes, USA). *E. coli* DH5α was used for plasmid constructions and was cultivated in LB medium [49] at 37 °C. Where appropriate, kanamycin (*E. coli* 50 µg/mL, *C. glutamicum* 25 µg/mL) was added to the medium. Bacterial growth was followed by measuring the optical density at 600 nm (OD_600_).

**Table 1:** Strains and plasmids used in this study

Strain or plasmid	Characteristics	Source
*C. glutamicum* strains		
DelAro^4^-*4cl*_*Pc*_ C7	*C. glutamicum* derivative with in-frame deletions of cg0344-47, cg0502, cg1226 and cg2625-40, harboring a chromosomally encoded codon-optimized *4cl*_*Pc*_ gene coding for 4-coumarate:CoA ligase from *Petroselinum crispum* under control of the T7 promoter (Δcg0344-47::P_T7_-*4cl*_*Pc*_) and replacement of the native *gltA* promoter with the *dapA* promoter variant C7 (P_*gltA*_::P_*dapA*_-C7)	[15]
Nor1 C7	DelAro^4^-*4cl*_*Pc*_ C7 strain harboring pMKEx2-*pcs*_*AaCg*_	This work
Nor2 C7	DelAro^4^-*4cl*_*Pc*_ C7 strain harboring pMKEx2-*pcs*_*AaCg*_-short	This work
Nor2 C7 mu*fasO*_*BC*_	Nor2 C7 derivative with mutated *fasO* binding site upstream of *accBC*	This work
Nor2 C7 mu*fasO*_*D1*_	Nor2 C7 derivative with mutated *fasO* binding site upstream of *accD1*	This work
Nor2 C7 mu*fasO*_*BCD1*_	Nor2 C7 derivative with mutated *fasO* binding site upstream of *accBC* and *accD1*	This work
Nor2 C5 mu*fasO*_*BCD1*_	Nor2 C7 mu*fasO*_*BCD1*_ derivative with replacement of the *dapA* promoter variant C7 with the *dapA* promoter variant C5	This work
Nor2 C5 mu*fasO*_*BCD1*_ P_O6_-*iolT1*	Nor2 C5 mu*fasO*_*BCD1*_ derivative with two nucleotide exchanges in the *iolT1* promoter at position -113 (A→G) and -112 (C→G) relative to the start codon	This work
Nor2 C5 mu*fasO*_*BCD1*_ P_O6_-*iolT1* ∆*pyc*	Nor2 C5 mu*fasO*_*BCD1*_ P_O6_-*iolT1* derivative with in-frame deletion of *pyc*	This work
*E. coli* strains		
DH5α	F–Φ80 *lacZ*ΔM15 Δ(*lacZYA*-*argF*)U169 *recA1 endA1 hsdR*17 (rK–,mKþ)*phoA supE*44λ–*thi*-*1 gyrA*96*relA1*	Invitrogen (Karlsruhe, Germany)
Plasmids		
pK19*mobsacB*	*kan*^r^; vector for allelic exchange in *C. glutamicum* (pK18, oriV_*Ec*_, *sacB*, *lac*Zα)	[52]
pK19*mobsacB*-mu*fasO*_*BC*_	Vector for mutation of the *fasO* binding site upstream of *accBC*	This work
pK19*mobsacB*-mu*fasO*_*D1*_	Vector for mutation of the *fasO* binding site upstream of *accD1*	This work
pK19*mobsacB*-P_*gltA*_::P_*dapA*_-C5	Vector for exchanging the P_*dapA*_-C7 variant upstream of *gltA* against the P_*dapA*_-C5 variant	[28]
pK19*mobsacB*-P_O6_-*iolT1*	Vector for mutation of the *iolT1* promoter	[29]
pK19*mobsacB*-∆*pyc*	Vector for in-frame deletion of *pyc*	This work
pMKEx2	*kan*^r^; *E. coli*-*C. glutamicum* shuttle vector (*lacI*, P_T7_, *lacO1*, pHM1519 ori_*Cg*_; pACYC177 ori_*Ec*_)	[24]
pMKEx2-*pcs*_*AaCg*_	pMKEx2 derivative with gene coding for pentaketide chromone synthase from *Aloe arborescens* (codon-optimized)	This work
pMKEx2-*pcs*_*AaCg*_-short	pMKEx2 derivative with truncated gene coding for pentaketide chromone synthase from *Aloe arborescens* (codon-optimized)	This work

To cultivate *C. glutamicum*, a test tube with 5 mL BHI medium was inoculated with a single colony from an agar plate and grown for 6–8 h on a rotary shaker at 170 rpm (first preculture). This first preculture was used to inoculate 50 mL of defined CGXII medium with 4% glucose in a 500 mL baffled Erlenmeyer flask (second preculture). The second preculture was cultivated overnight on a rotary shaker at 130 rpm. The main culture was subsequently inoculated from the second preculture to an OD_600_ of 5 in defined CGXII medium with 4% glucose. For synthesis of noreugenin, heterologous gene expression was induced 90 min after inoculation of the main culture using 1 mM IPTG. 1 mL of the culture broth was sampled at defined time points and stored at − 20 °C until ethyl acetate extraction and LC–MS analysis.

### Plasmid and strain construction

Standard protocols of molecular cloning, such as PCR, restriction and ligation of DNA were carried out for recombinant DNA work [50]. All enzymes were obtained from Thermo Fisher Scientific (Schwerte, Germany). Codon-optimized synthetic genes for *C. glutamicum* ATCC13032 were obtained from Life Technologies (Darmstadt, Germany). Genes and chromosomal fragments were amplified by PCR from synthetic genes or genomic *C. glutamicum* DNA as template using the listed oligonucleotides (Table 2). PCR products were subsequently used for cloning of genes and genomic fragments into plasmid vectors using Gibson Assembly [51]. In-frame gene deletions and introduction of genomic mutations in *C. glutamicum* were performed using the pK19mobsacB system [52] by a two-step homologous recombination method described previously [53]. Integrity of all constructed plasmids was verified by colony PCR, restriction analysis, and DNA sequencing at Eurofins MWG Operon (Ebersberg, Germany) Techniques specific for *C. glutamicum*, e.g. electroporation of cells, were performed as described previously [54].

**Table 2:** Oligonucleotides used in this study

Primer	Sequence (5′– > 3′)
PCS-s	ACTTTAAGAAGGAGATATACCATGGTAAGGAGGACAGCTATGTCCTCCTTGTCCAAC
PCS-as	CCAGGACTAGTTTCCAGAGTACTATTACATGAGTGGCAGGGAG
PCS-short-s	ACTTTAAGAAGGAGATATACCATGGTAAGGAGGACAGCTATGGAAGATGTGCAGGGC
mu-accBC-up-s	ATCCCCGGGTACCGAGCTCGAACCAGCGCGCGTTCGTG
mu-accBC-up-as	TTACGACTATTCTGGGGGAATTCTTCTGTTTTAGGCAGGAAATATGGCTTATG
mu-accBC-down-s	AGAAGAATTCCCCCAGAATAGTCGTAAGTAAGCATATCTGGTTGAGTTCTTCGGGGTTG
mu-accBC-down-as	TTGTAAAACGACGGCCAGTGGCCTTGGCGGTATCTGCG
chk-accBC-s	GTTCGGCCACTCCGATGTCCGCCTG
chk-accBC-as	GCCTTGATGGCGATTGGGAGACC
mu-accD1-up-s	ATCCCCGGGTACCGAGCTCGTCATTCAACGCATCCATGACAGC
mu-accD1-up-as	CTAATGGTCATGTTTTGAAATCGTAGCGGTAGGCGGGG
mu-accD1-down-s	ACCGCTACGATTTCAAAACATGACCATTAGTAGCCCTTTGATTGACGTCGCCAACCTTC
mu-accD1-down-as	TTGTAAAACGACGGCCAGTGCGCCAGAAGCCTGAATGTTTTG
chk-accD1-s	GGCTGATATTAGTGCCCCAACCGATGAC
chk-accD1-as	GATCACGTCTGGGCCGGTAACGAAC
chk-gltA-s	ATCGTTAACGATCTGACCCAACAA
chk-gltA-as	CGTAAGCAGCCTCTGGCGGAA
chk-P_O6_-iolT1-s	TACGAATGCCCACTTCGCACCCTT
chk-P_O6_-iolT1-as	CAACTCATTACGGCCAGCCAGTGAGC
pyc-up-s	ATCCCCGGGTACCGAGCTCGAATTCCTGATACCTTCGCGGTGTAC
pyc-up-as	CACCTTCCACAGATGTGTGAGTCGACAC
pyc-down-s	TCACACATCTGTGGAAGGTGGCGACTTG
pyc-down-as	TTGTAAAACGACGGCCAGTGAATTCCCTGAAAGTGCAGAATGTCTTTTTC
chk-pyc-s	GCCGTAACTCTGGCCTGATC
chk-pyc-as	CTGGCAACCACATCTGCACTGCG
chk-pMKEx2-s	CCCTCAAGACCCGTTTAGAGGC
chk-pMKEx2-as	TTAATACGACTCACTATAGGGGAATTGTGAGC
rsp	CACAGGAAACAGCTATGACCATG
univ	CGCCAGGGTTTTCCCAGTCACGAC

### Noreugenin extraction and LC–MS quantification

Noreugenin extraction was performed by mixing 1 mL of the culture broth with 1 mL ethyl acetate and subsequent vigorous shaking (1400 rpm, 10 min, 20 °C) in a thermomixer (Eppendorf, Hamburg, Germany). The suspension was centrifuged for 5 min at 13,300 rpm and the upper, organic layer (800 µL) was transferred to an organic solvent resistant deep-well plate (Eppendorf, Hamburg, Germany). After evaporation of the ethyl acetate overnight, the same volume of acetonitrile was used to resuspend the dried extracts for LC–MS analysis. Noreugenin was quantified using an Agilent ultra-high-performance LC (uHPLC) 1290 Infinity System coupled to a 6130 Quadrupole LC–MS System (Agilent Technologies, Waldbronn, Germany). LC separation was carried out with a Kinetex 1.7 µm C_18_ 100 Å pore size column (2.1 * 50 mm; Phenomenex, Torrance, CA, USA) at 50 °C. For elution, 0.1% acetic acid (solvent A) and acetonitrile supplemented with 0.1% acetic acid (solvent B) were applied as the mobile phases at a flow rate of 0.5 mL/min. A gradient elution was used, where the amount of solvent B was increased stepwise: minute 0 to 6: 10% to 30%, minute 6 to 7: 30% to 50%, minute 7 to 8: 50% to 100% and minute 8 to 8.5: 100% to 10%. The mass spectrometer was operated in the negative electrospray ionization (ESI) mode, and data were acquired using the selected ion monitoring (SIM) mode. An authentic noreugenin standard was purchased from Carbosynth (Compton, Newbury, United Kingdom). Area values for [M−H]^−^ mass signals were linear up to metabolite concentrations of at least 50 mg/L. Benzoic acid (final concentration 100 mg/L) was used as internal standard. A calibration curve was calculated based on analyte/internal standard ratios for the obtained area values.

### Acid-catalyzed cyclization of 1-(2,4,6-trihydroxyphenyl)butane-1,3-dione

For the cyclization of 1-(2,4,6-trihydroxyphenyl)butane-1,3-dione, a 250 µL aliquot of the ethyl acetate-extracted acetonitrile samples prepared for LC–MS was acidified by addition of 100 µL 0.5 M HCl in MeOH (final concentration 0.15 M). The samples were incubated (700 rpm, 3 h, 50 °C) in a thermomixer (Eppendorf, Hamburg, Germany), evaporated to dryness and subsequently resuspended in 250 µL acetonitrile. LC–MS analysis of obtained samples was performed as described above.

## Additional file

**Additional file 1.** Additional information containing detailed alignment of amino acid sequences, further results and a proposed mechanism for the acid-catalyzed cyclization of TPBD.
