# Supplementary material for: Tailoring Corynebacterium glutamicum towards increased malonyl-CoA availability for efficient synthesis of the plant pentaketide noreugenin
Source: Microb Cell Fact. 2019 Apr 11;18:71. doi: 10.1186/s12934-019-1117-x (PMC6460773; doi:10.1186/s12934-019-1117-x)
Supplement: Supplementary file 1 — Additional file 1. Additional information containing detailed alignment of amino acid sequences, further results and a proposed mechanism for the acid-catalyzed cyclization of TPBD. [file 12934_2019_1117_MOESM1_ESM.pdf]

Additional file 1

Tailoring *Corynebacterium glutamicum* towards increased malonyl-CoA availability for efficient synthesis of the plant pentaketide noreugenin

Lars Milke[1], Nicolai Kallscheuer[1,2], Jannick Kappelmann[1], Jan Marienhagen[1,2,3 *]

[1]Institute of Bio- and Geosciences, IBG-1: Biotechnology, Forschungszentrum Jülich, 52425 Jülich, Germany

[2]Bioeconomy Science Center (BioSC), Forschungszentrum Jülich GmbH, 52425 Jülich, Germany

[3]Institute of Biotechnology, RWTH Aachen University, Worringer Weg 3, 52074 Aachen, Germany

[*] Corresponding author:

Prof. Dr. Jan Marienhagen, phone +49 2461 61 2843, e-mail j.marienhagen@fz-juelich.de

Keywords: malonyl-CoA, *Corynebacterium glutamicum*, noreugenin, metabolic engineering, acetyl-CoA carboxylase

Additional file 1

```
PCS_Aa (Q58VP7)    MSSLSNSLPLMEDVQGIRKAQKADGTATVMAIGTAHPPHIFPQDTYADVYFRATNSEHKV 60
STS_Ah (K7XD27)    ----------MVSVSGIRNVERAEGPATVLAIGTANPSNCVDQSTYADYYFRVTNSEHMT 50
CHS_Ph (Q43040)    ----------MVTVEEYRKAQRCEGPATVMAIGTATPTNCVDQSTYPDYYFRITNSEHKT 50
                             *  *.  *:.::.:* ***:***** * : . *.** * *** ***** .

PCS_Aa (Q58VP7)    ELKKKFDHICKKTMIGKRYFNYDEEFLKKYPNITSYDEPSLNDRQDICVPGVPALGTEAA 120
STS_Ah (K7XD27)    DLKKKFQRICERTQIKNRHMYLTEEILKENPNICAYKAPSLDAREDMMIREVPRVGKEAA 110
CHS_Ph (Q43040)    DLKEKFKRMCEKSMIKKRYMHLTEEILKENPSMCEYMAPSLDARQDIVVVEVPKLGKEAA 110
                   :**:**.::*::: * :*::   **:**: *.:  *  ***: *:*: :  ** :*.***

PCS_Aa (Q58VP7)    VKAIEEWGRPKSEITHLVFCTSCGVDMPSADFQCAKLLGLHANVNKYCIYMQGCYAGGTV 180
STS_Ah (K7XD27)    TKAIKEWGQPMSKITHLIFCTTSGVALPGVDYELIVLLGLDPCVKRYMMYHQGCFAGGTV 170
CHS_Ph (Q43040)    QKAIKEWGQPKSKITHLVFCTTSGVDMPGCDYQLTKLLGLRPSVKRLMMYQQGCFAGGTV 170
                    ***:***:* *:****:***:.** :*. *::    ****   *::  :* ***:*****

PCS_Aa (Q58VP7)    MRYAKDLAENNRGARVLVVCAELTIMMLRAPNETHLDNAIGISLFGDGAAALIIGSDPII 240
STS_Ah (K7XD27)    LRLAKDLAENNKDARVLIVCSENTAVTFRGPSETDMDSLVGQALFADGAAAIIIGSDPVP 230
CHS_Ph (Q43040)    LRLRKDLAENNKGARVLVVCSEITAVTFRGPNDTHLDSLVGQALFGDGAGAIIIGSDPIP 230
                   :*  *******:.****:**:* * : :*.*.:*.:*. :* :**.***.*:******:

PCS_Aa (Q58VP7)    GVEKPMFEIVCTKQTVIPNTEDVIHLHLRETGMMFYLSKGSPMTISNNVEACLIDVFKSV 300
STS_Ah (K7XD27)    EVEKPLFEIVSTDQKLVPGSHGAIGGLLREVGLTFYLNKSVPDIISQNINDALSKAFDPL 290
CHS_Ph (Q43040)    GVERPLFELVSAAQTLLPDSHGAIDGHLREVGLTFHLLKDVPGLISKNIEKSLEEAFKPL 290
                    **:*:**:*.: *.::*.:...*   ***.*: *:* *. *  **:*:: .* ..*. :

PCS_Aa (Q58VP7)    GITPPEDWNSLFWIPHPGGRAILDQVEAKLKLRPEKFRAARTVLWDYGNMVSASVGYILD 360
STS_Ah (K7XD27)    GI---SDYNSIFWIAHPGGRAILDQVEQKVNLKPEKMKATRDVLSNYGNMSSACVFFIMD 347
CHS_Ph (Q43040)    GI---SDWNSLFWIAHPGGPAILDQVEIKLGLKPEKLKATRNVLSDYGNMSSACVLFILD 347
                   **   .*:**:*** **** ******* *: *:***::*:* ** :**** **.* :*:*

PCS_Aa (Q58VP7)    EMRRKSAAKGLETYGEGLEWGVLLGFGPGITVETILLHSLPLM 403
STS_Ah (K7XD27)    LMRKKSLEEGLKTTGEGLDWGVLFGFGPGLTIETVVLRSVAI- 389
CHS_Ph (Q43040)    EMRKASAKEGLGTTGEGLEWGVLFGFGPGLTVETVVLHSVAT- 389
                    **: *  :** * ****:****:*****:*:**::*:*:
```

Figure S1: Alignment of the amino acid sequences of the pentaketide chromone synthase from *Aloe arborescens* (PCS$_{Aa}$, UniProt ID Q58VP7), the stilbene synthase from *Arachis hypogea* (STS$_{Ah}$, UniProt ID K7XD27) and the chalcone synthase from *Petunia x hybrida* (CHS$_{Ph}$, UniProt ID Q43040). The ten N-terminal amino acids of PCS$_{Aa}$ which were deleted in the course of this study are highlighted by the orange box.

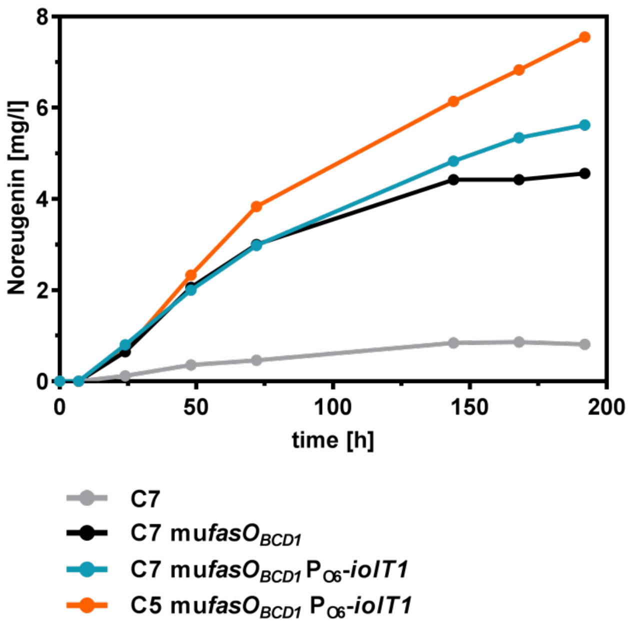

Figure S2: Microbial production of noreugenin from glucose during shake flask cultivations with engineered *C. glutamicum* strains harboring pMKEx2-*pcs*$_{AaCg}$-short during extended cultivation times. The data represent values from single replicate experiments.

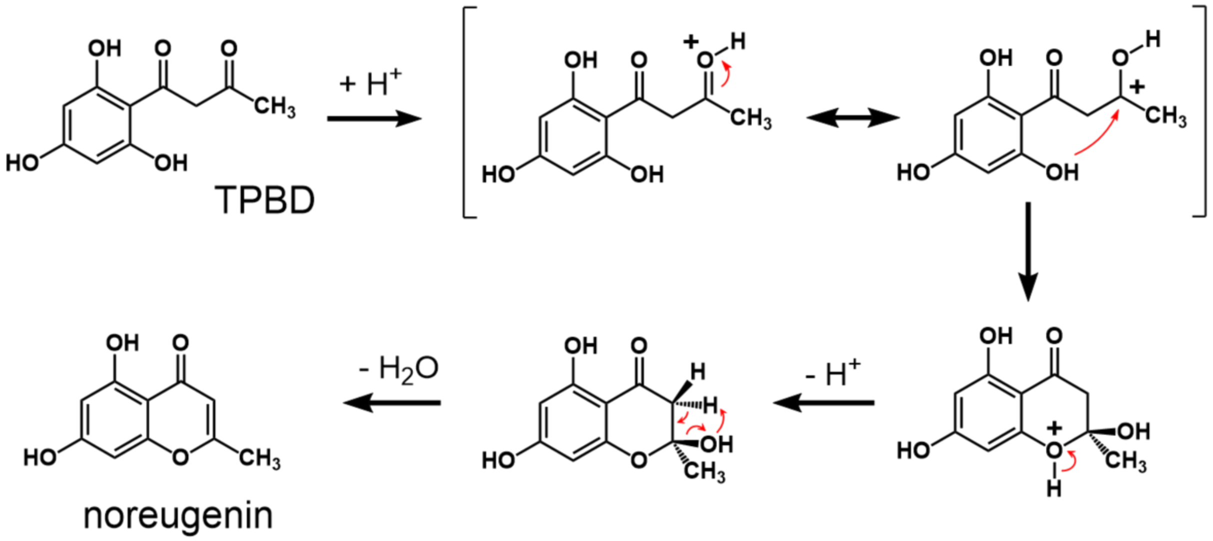

Figure S3: Proposed mechanism for the HCl-catalyzed cyclization of the intermediate 1-(2,4,6-trihydroxyphenyl)butane-1,3-dione (TPBD) yielding noreugenin.
